# The value of preoperative diagnostic testing and geriatric assessment in frail institutionalized elderly with a hip fracture; a secondary analysis of the FRAIL-HIP study

**DOI:** 10.1007/s41999-024-00945-8

**Published:** 2024-02-28

**Authors:** Miliaan L. Zeelenberg, Dennis Den Hartog, Esther M. M. Van Lieshout, Hugo H. Wijnen, Hanna C. Willems, Taco Gosens, Jeroen Steens, Romke Van Balen, Rutger G. Zuurmond, Sverre A. I. Loggers, Pieter Joosse, Michael H. J. Verhofstad, FRAIL-HIP Investigator Group

**Affiliations:** 1 Trauma Research Unit, Department of Surgery, Erasmus MC, University Medical Center Rotterdam, P.O. Box 2040, 3000 CA Rotterdam, The Netherlands; 2 Department of Clinical Geriatrics, Rijnstate Hospital, Arnhem, The Netherlands; 3 Department of Internal Medicine and Geriatrics, Amsterdam University Medical Center, Amsterdam, The Netherlands; 4 Department of Orthopedics, Elisabeth Hospital (ETZ), Tilburg, The Netherlands; 5 Department of Surgery, Dijklander Hospital, Hoorn, The Netherlands; 6 Department of Public Public Health and Primary Care, Leiden University Medical Center, Leiden, The Netherlands; 7 Department of Orthopedics, Isala Hospital, Zwolle, The Netherlands; 8 Department of Surgery, Noordwest Ziekenhuisgroep, Alkmaar, The Netherlands

**Keywords:** Frail older adults, Hip fracture, Screening, Diagnostics, (non-)operative treatment

## Abstract

**Purpose:**

The aim of this study was to provide a comprehensive overview of (preoperative and geriatric) diagnostic testing, abnormal diagnostic tests and their subsequent interventions, and clinical relevance in frail older adults with a hip fracture.

**Methods:**

Data on clinical consultations, radiological, laboratory, and microbiological diagnostics were extracted from the medical files of all patients included in the FRAIL-HIP study (inclusion criteria: hip fracture, > 70 years, living in a nursing home with malnourishment/cachexia and/or impaired mobility and/or severe co-morbidity). Data were evaluated until hospital discharge in nonoperatively treated patients and until surgery in operatively treated patients.

**Results:**

A total of 172 patients (88 nonoperative and 84 operative) were included, of whom 156 (91%) underwent laboratory diagnostics, 126 (73%) chest X-rays, and 23 (13%) CT-scans. In 153/156 (98%) patients at least one abnormal result was found in laboratory diagnostics. In 82/153 (50%) patients this did not result in any additional diagnostics or (pharmacological) intervention. Abnormal test results were mentioned as one of the deciding arguments for operative delay (> 24 h) for 10/84 (12%) patients and as a factor in the decision between nonoperative and operative treatment in 7/172 (4%) patients.

**Conclusion:**

A large number and variety of diagnostics were performed in this patient population. Abnormal test results in laboratory diagnostics were found for almost all patients and, in majority, appear to have no direct clinical consequences. To prevent unnecessary diagnostics, prospective research is required to evaluate the clinical consequences and added value of the separate elements of preoperative diagnostic testing and geriatric assessment in frail hip fracture patients.

**Supplementary Information:**

The online version contains supplementary material available at 10.1007/s41999-024-00945-8.

## Key summary points

**Aim:**

This study evaluated (preoperative and geriatric) diagnostic testing and its consequences in operatively and nonoperatively treated frail older adults with an acute hip fracture.

**Findings:**

A large number and variety of diagnostics were performed in this patient population. Abnormal test results in laboratory diagnostics were found for almost all patients and, in majority, appear to have no direct clinical consequences or influence on the treatment choice for the hip fracture.

**Message:**

To prevent unnecessary diagnostics, prospective research is required to evaluate the clinical consequences and added value of the separate elements of preoperative diagnostic testing and geriatric assessment in frail hip fracture patients.

**Supplementary Information:**

The online version contains supplementary material available at 10.1007/s41999-024-00945-8.

## Introduction

A proximal femoral fracture in frail institutionalized patients is associated with high mortality and significantly diminished health-related quality of life [1–4]. The results of the FRAIL-HIP study suggested that nonoperative care is a viable option in the most frail older adults with a hip fracture [5]. Patients who opted for nonoperative management showed a reduced life expectancy, but their quality of life was not inferior to operated patients and the dying process without surgery was judged humane [5, 6].

When these patients are admitted with a suspected hip fracture, an extensive diagnostic process is started. In addition to regular radiographs and physical examination, the Dutch guideline for surgical treatment of frail older adults recommends a geriatric assessment in addition to the general preoperative screening [7]. In frail older adults with multiple pre-trauma comorbidities, many incidental abnormalities can be expected during the diagnostic process. Often clinical findings at arrival warrant additional laboratory diagnostics, chest X-rays, or cardiac ultrasound, and in some cases extensive routine testing is performed as standard care.

Questions could be raised about whether these abnormalities have consequences for patients. Many abnormalities found through routine preoperative diagnostic testing have little predictive value for outcomes in surgical patients [8–10]. It is unknown what proportion of abnormal diagnostic tests requires additional interventions and whether these are performed. Also, the influence of preoperative assessment and diagnostic testing on the treatment choice (operative versus nonoperative) is unclear. Studies on preoperative cardiac testing show no changes in the perioperative management of older adults with a hip fracture, whereas testing significantly increases the costs of care and/or time to surgery [11–13]. As less than one percent of routine preoperative chest radiographs in patients with hip fractures lead to a clinically significant finding, some authors advocate only selective use based on clinical indicators instead of routine testing [14, 15]. Preventing unnecessary preoperative diagnostic testing could be vital in reducing the burden of care for admitted patients, delay of surgery, and general costs of care.

Few data exist on (preoperative) diagnostic testing and its associated interventions in frail older adults with a proximal femoral fracture. The aim of this study was to provide a comprehensive overview of (routine) preoperative and geriatric diagnostic testing, and the number of associated interventions with regards to frail institutionalized older adults with a proximal femoral fracture.

## Methods

This retrospective cohort study conducted an additional analysis of patients who participated in the FRAIL-HIP study [5]. These patients were enrolled between September 1, 2018 and April 25, 2020.

The FRAIL-HIP study was a prospective cohort study performed in 25 hospitals across the Netherlands. Eligible patients were aged 70 years or older, frail, institutionalized, and sustained a femoral neck or trochanteric fracture. The term frail implied that at least one of the following characteristics was present: malnutrition (body mass index < 18.5 kg.m^2^), severe comorbidities (American Society of Anesthesiologists physical status class of IV or V), or severe mobility issues (Functional Ambulation Category ≤ 2). The Functional Ambulation Categories (FAC) is a functional walking test that evaluates ambulation ability [16]. This 6-point scale assesses ambulation status by determining how much human support the patient requires when walking, regardless of whether or not they use a personal assistive device. FAC ≤ 2 means that patients at least have the need for (intermittent) help of another person to be able to ambulate (FAC 2) to no functional ambulatory capabilities (FAC 0).

Each patient was either treated operatively or nonoperatively. Treatment decision was made by shared decision making based on the personal preference and treatment goals of patients and/or relatives. Patients were followed until six months after trauma or until death.

### Outcome measures

The primary outcome of this post hoc analysis was the type and number of diagnostic tests performed as part of in-hospital (preoperative or geriatric) assessment. This was categorized into: consultations, radiological diagnostics and electrocardiograms (ECGs), laboratory (blood) and microbiological diagnostics. Secondary outcomes were: abnormal results found during testing, interventions or additional diagnostics initiated after abnormal results during diagnostic testing, postponement of surgery due to abnormal diagnostic test results, change in hip fracture treatment due to abnormal diagnostic test results, and adherence to the national guidelines with regards to diagnostic testing and preoperative assessment in frail older adults with a proximal femur fracture. Data on diagnostic procedures, test results, and subsequent interventions were either extracted from the FRAIL-HIP database or extracted from the patients’ hospital records. For operatively treated patients, data were extracted until the moment of operation and for conservatively treated patients until discharge from the hospital. All data were collected as reported in hospital records; no new interpretations or test results were created based on available test data. Results were classified as an abnormal test result if mentioned as such in the patients’ records or classified as abnormal in the test registration. Abnormal diagnostic test results were classified as known if abnormal test results of similar magnitude were found in recent tests preceding the hip fracture admission, or if a related (chronic) disease was recorded in their recent medical history. Consequences of abnormal diagnostic tests were classified as no diagnostics or intervention (including expectant/conservative treatment or no mention of additional diagnostics or intervention), additional diagnostic testing (including all types of diagnostics related to the abnormal result or repeated measurement of the same test), pharmacological intervention (related starting, altering or stopping of medication), or invasive intervention (related surgical intervention or other non-diagnostic invasive procedures). Pain medication was not registered as additional intervention since all patients used analgesic medication in this study. Diagnostics were classified as indicated if they were performed after an explicit report of a complaint or clinical indication in the patient’s file. In all other cases they were defined as screening diagnostics. Additional data on the patients’ vital signs and physical state at admission were gathered, including heart rate, blood pressure, respiratory rate, oxygen saturation, neurological status, and abnormalities found during physical examination.

In addition, the proportion of conducted laboratory diagnostics in accordance with the advised laboratory tests by the Dutch guidelines was assessed [7, 17]. The Dutch hip fracture guideline advises the minimum preoperative laboratory diagnostics to contain at least: hemoglobin, albumin, creatinine/eGFR, electrolytes (most likely represented by sodium, potassium, and calcium), and glucose [7]. In addition, the Dutch guideline for the comprehensive geriatric assessment advises an assessment of: erythrocyte sedimentation rate (ESR), complete blood count, kidney function, glucose, thyroid assessment, electrolytes, liver enzymes, Vitamin B12, folic acid, total serum protein and albumin [17].

These data were combined with the pre-existing FRAIL-HIP database, including patient characteristics (age, sex, body mass index, ASA classification, Charlson comorbidity index), type of fracture, additional injuries, and treatment (operative versus nonoperative).

### Statistical analysis

Data were analyzed using the Statistical Package for the Social Sciences (SPSS) version 28.0 (SPSS, Chicago, Ill., USA). Normality for continuous data was assessed using the Shapiro–Wilk test. The analyses were done stratified for nonoperative treatment and operative treatment. Continuous data were reported as mean and SD (if normally distributed) or median and interquartile ranges (in case of non-normal distribution) and categorical data as numbers with percentages. Univariate comparison between groups was performed using a Mann–Whitney, χ2, or Fischer exact tests. A 2-sided *P*-value < 0.05 was used as threshold for statistical significance.

## Results

A total of 172 patients with a hip fracture were included, of which 88 were treated nonoperatively, and 84 were treated operatively. No significant differences were found in baseline characteristics, physical parameters, or findings during physical examination between the two treatment groups (Table 1; Online Resource 1 (OR 1), Table S1). Consultations, radiological diagnostics, laboratory diagnostics, and microbiological diagnostics were mainly requested at the emergency department (> 90%; OR 1, Tables S2–S3). Radiological diagnostics were primarily performed after a clinical indication (*e.g.*, head trauma or painful wrist after a fall), whereas laboratory and microbiology diagnostics were mostly performed without mention of a specific clinical indication or related disease (OR 1, Tables S4–S5).

**Table 1:** Baseline characteristics for non-operatively and operatively treated patients

	Nonoperative (*n* = 88)		Operative (*n* = 84)		*P*-value
	N*		N*		
Women	88	67 (76%)	84	68 (81%)	0.28
BMI (kg/m^2^)	68	20.4 (18.0–25.2)	77	23.2 (18.4–26.3)	0.10
*ASA classification*	88		84		0.41
2		4 (5%)		1 (1%)	
3		53 (60%)		54 (64%)	
4		31 (35%)		29 (35%)	
CCI	88	3 (2–5)	84	3 (2–5)	0.44
*Type of fracture*					
Femoral neck	88	54 (61%)	84	45 (54%)	0.30
Trochanteric		34 (39%)		39 (46%)	0.94
*Physical parameters at admission*					
Temperature (ºC)	75	37.0 (SD 0.56)	79	36.9 (SD 0.66)	0.14
Heart rate (bpm)	81	81 (SD 15)	81	81 (SD 15)	0.14
SBP (mmHg)	81	149 (SD 30)	83	149 (SD 28)	0.94
DBP (mmHg)	81	78 (SD 15)	83	78 (SD 16)	0.90
O_2_ saturation (%)	80	95 (94–97)	79	95 (93–97)	0.29
Respiratory rate	53	15 (14–19)	68	16 (14–16)	0.95
GCS	34	15 (14–15)	48	15 (15–15)	0.18

Data are shown as *n* (%), median (P_25_-P_75_) or mean (SD)

N* refers to the number of patients for whom data were available

*ASA* American school of Anesthesiologists, *BMI* body mass index, *bpm* beats per minute, *CCI* Charlson comorbidity score, *DBP* diastolic blood pressure, *GCS* Glasgow coma scale, *SBP* systolic blood pressure

### Clinical consultations

The most common clinical consultations (Table 2), excluding (orthopedic) surgery consultations (100% in both arms), were geriatric (59/88 nonoperative versus 77/84 operative) and cardiology (8/88 nonoperative versus 22/84 operative) consultations. No additional clinical consultations occurred in 28/88 nonoperative patients and in only 1/84 patients in the operative group (*p* < 0.001). Seventeen of those 28 nonoperative patients without consultations also received no other diagnostics than a pelvic X-ray at the ER and directly returned to their nursing home after having chosen nonoperative treatment. Both geriatric and cardiology consultations occurred significantly more in the operative group. After geriatric consultations, 39/59 (66%) nonoperative and 19/77 (25%) operative patients had no (pharmacological) intervention or additional diagnostics (OR1, Table S6). For cardiology consultations, this was 2/8 (25%) for nonoperative patients and 7/22 (32%) for operative patients. No patients received invasive interventions due to geriatric or cardiologic consultations.

**Table 2:** Clinical consultations for non-operatively and operatively treated patients

Consulted specialism	Nonoperative (*n* = 88)	Operative (*n* = 84)
(Orthopedic) Surgery	88 (100%)	84 (100%)
Geriatrics	59 (67%)*	77 (92%)
Cardiology	8 (9%)*	22 (26%)
Neurology	2 (2%)	4 (5%)
Internal medicine	1 (1%)	3 (3%)
Pulmonary medicine	2 (2%)	1 (1%)
Anesthesia^a^	2 (2%)	0 (0%)
Other^b^	1 (1%)	4 (5%)
No consultation^c^	28 (32%)*	1 (1%)

Data are shown as *n* (%). *Statistically significant difference (*p* < 0.05) between nonoperative and operative groups

^a^Not being regular preoperative screening consultation

^b^Other including: Psychiatry, Microbiology, ophthalmology, urology

^c^No consultations other than primary orthopedic or (trauma-)surgery consultation

### Radiological diagnostics and electrocardiograms

As a radiologically proven cervical neck or trochanteric fracture was a requirement for inclusion, all patients received a form of radiological diagnostics (Table 3). Other than pelvic X-rays, chest X-rays were performed in 59/88 (67%) nonoperative patients and 67/84 (80%) operative patients. In 41/59 nonoperative and 32/67 operative patients, abnormal results were found on the chest X-ray. The most common abnormalities found were cardiomegaly (18/59 versus 15/67) and pulmonary consolidation/suspected infection (8/59 versus 8/67) (OR 1, Table S7). In 18/59 nonoperative patients and 35/67 operative patients, no abnormal results were found in chest X-rays; this was significantly more in the operative group (*p* = 0.011). Other types of X-rays were less common, with arm/wrist X-rays (six nonoperative and six operative) and knee X-rays (three nonoperative and five operative) being most frequent. CT-scans were made in nine nonoperative and 14 operative patients, with head and cervical spine as the most common categories.

**Table 3:** Radiological diagnostics and electrocardiograms (ECGs) performed and abnormal diagnostic results found

	Nonoperative (*n* = 88)			Operative (*n* = 84)		
	Performed	Abnormal test results	Additional diagnostics /Intervention	Performed	Abnormal test results	Additional diagnostics /Intervention
*X-rays*	88 (100%)			84 (100%)		
Pelvis^a^	88 (100%)	87 (98%)	2 (2%)*	84 (100%)	84 (100%)	84 (100%)
Thorax	59 (67%)	41 (69%)*	5 (12%)	67 (80%)	32 (48%)	5 (16%)
Shoulder/clavicle^b^	0 (0%)	N.A	N.A	3 (4%)	2	0
Arm/wrist^c^	6 (7%)	5	1	6 (7%)	4	2
Hand	0 (0%)	N.A	N.A	1 (1%)	1	0
Upper leg	2 (2%)	0	0	0 (0%)	N.A	N.A
Knee	3 (3%)	0	0	5 (6%)	3	0
Lower leg	1 (1%)	1	0	1 (1%)	0	0
*CT-scans*	9 (10%)			14 (17%)		
Pelvis	2 (2%)	2	0	6 (7%)	6	5
Thorax	1 (1%)	1	0	1 (1%)	1	0
Cervical spine	4 (5%)	3	0	6 (7%)	4	0
Head	8 (9%)	5	0	8 (10%)	6	0
Face	1 (1%)	0	0	0 (0%)	N.A	N.A
*MRI-scans*	0 (0%)	N.A	N.A	0 (0%)	N.A	N.A
*Ultra-sound*	4 (5%)*			14 (17%)		
Cardiac	4 (5%)*	4	0	14 (17%)	10	0
Abdominal	1 (1%)	1	0	0 (0%)	N.A	N.A
*ECG*^d^	47 (53%)*	32 (68%)	1 (3%)	69 (82%)	41 (59%)	3 (7%)

Data are shown as *n* (%). *Statistically significant difference (*p* < 0.05) between nonoperative and operative groups

^a^One operative patient had 3 pelvic X-rays and one operative patient had 2 pelvic X-rays

^b^One operative patient had 2 shoulder/clavicle X-rays

^c^One operative patient had 2 arm/wrist X-rays

^d^One nonoperative patient had 2 ECGs

N.A., not applicable

Cardiac ultrasounds were performed significantly less (*p* = 0.12) in nonoperative patients than in operative patients (4 with 4 abnormal test versus 14 with 10 abnormal tests). Electrocardiography was also performed more often in the operative group (47 versus 69, *p* < 0.001), with 32 and 41 abnormal tests found, of which most were previously known abnormalities, respectively, 21 and 26.

Regarding consequences (OR 1, Table S8), radiological diagnostics or ECGs rarely led to additional diagnostics or interventions in the nonoperative group, only six pharmacological or invasive interventions across all diagnostic tests. Likewise, in the operative group (besides hip fracture surgery) a total of 13 interventions were conducted related to performed radiological diagnostics.

### Laboratory diagnostics

Laboratory diagnostics (Table 4) were performed in 72 (82%) of nonoperative patients and 84 (100%) of operative patients (*p* < 0.001). All lab categories and microbiological tests, except for liver/pancreas and cardiac function, were performed significantly more in the operative group. In only one patient in the nonoperative group and two patients in the operative group, no abnormal test results were found in any of the laboratory diagnostics. Hematological diagnostics was the only subgroup with significantly more abnormal test results in the nonoperative group (64/72 versus 62/84, *p* = 0.006). Most specific abnormal test results were found in hemoglobin (46/72 nonoperative and 36/84 operative), leukocyte count (45/71 nonoperative and 37/80 operative), Urea (37/65 nonoperative and 48/78 operative), and C-reactive protein (41/68 nonoperative and 48/80 operative). The most relevant tests and abnormalities, as described by the Dutch guideline for hip fracture treatment and guideline for CGA are shown in Table 5. Nonoperative patients had significantly lower values for hemoglobin (7.4 versus 7.8 mmol, *p* = 0.009), hematocrit (0.36 versus 0.38 mmol, *p* = 0.016), and serum albumin (34 versus 35 g/L, *p* = 0.023), but had a higher glomerular filtration rate (eGFR) than operative patients (65 versus 60 mL/min/1,73m^2^, *p* = 0.026). All other specific laboratory diagnostic results can be found in Online Resource 1 (Table S9).

**Table 4:** Laboratory diagnostics performed, abnormal test results found, and subsequent additional diagnostics or intervention performed per subcategory

	Nonoperative (*n* = 88)			Operative (*n* = 84)		
	Performed	Abnormal test results^a^	Additional diagnostics or Intervention	Performed	Abnormal test results^a^	Additional diagnostics or intervention
*Laboratory testing*						
Hematological	72 (82%)*	64 (89%)*	9 (14%)*	84 (100%)	62 (74%)	20 (32%)
Electrolytes	72 (82%)*	25 (65%)	4 (16%)	82 (98%)	32 (61%)	11 (34%)
Nutritional status	67 (76%)*	37 (55%)	2 (5%)	81 (96%)	46 (57%)	6 (13%)
Vitamin status	31 (35%)*	9 (29%)	1 (11%)*	49 (58%)	21 (43%)	13 (62%)
Inflammatory markers	69 (78%)*	42 (61%)	8 (19%)	80 (95%)	51 (64%)	18 (35%)
Kidney function	71 (81%)*	43 (61%)	3 (7%)	83 (99%)	56 (68%)	13 (23%)
Liver/pancreas function	61 (69%)*	37 (61%)	1 (3%)	68 (81%)	37 (54%)	2 (5%)
Cardiac function	2 (2%)	2 (100%)	1 (50%)	1 (1%)	1 (100%)	0 (0%)
(Para-)Thyroid function	37 (42%)*	10 (27%)	1 (10%)	54 (64%)	18 (33%)	0 (0%)
Coagulation	62 (71%)*	18 (29%)	10 (55%)	81 (96%)	19 (24%)	15 (79%)
Arterial blood gas	3 (3%)	2 (67%)	2 (100%)	1 (1.2%)	1 (100%)	0 (0%)
Urine sediment	22 (25%)*	6 (27%)	2 (33%)	46 (55%)	18 (39%)	14 (78%)
Microbiological testing	10 (11%)*			20 (24%)		
Blood culture	3	2	0	5	1	1
Urine culture	8	5	0	14	10	5
Viral PCR	1	0	N.A	2	0	N.A
MRSA culture	0	N.A	N.A	1	0	N.A
Other						
D-dimer	0	N.A	N.A	1	1	1

Data are shown as *n* (%). *Statistically significant difference (*p* < 0.05) between nonoperative and operative groups

^a^At least one abnormal test result in the whole subcategory

*N.A.* not applicable

**Table 5:** Number of patients receiving specific laboratory diagnostics and abnormal test results in the minimally required laboratory tests as described by Dutch hip fracture guideline and/or guideline for comprehensive geriatric assessment

	Nonoperative (*n* = 88)		Operative (*n* = 84)	
	Performed	Abnormal test results	Performed	Abnormal test results
Hemoglobin	72 (82%)*	46 (64%)*	84 (100%)	36 (43%)
eGFR	71 (81%)*	26 (37%)	83 (99%)	34 (41%)
Sodium	72 (82%)*	9 (13%)	82 (98%)	8 (10%)
Potassium	72 (82%)*	9 (13%)	81 (96%)	11 (14%)
Calcium	58 (66%)*	6 (10%)	68 (81%)	3 (4%)
Albumin	58 (66%)*	28 (48%)*	69 (82%)	20 (29%)
Glucose	66 (75%)*	15 (23%)*	80 (95%)	33 (41%)
*Additional tests mentioned in guideline for CGA*				
ESR	24 (27%)	8 (33%)	27 (32%)	11 (41%)
Hematocrit	68 (77%)*	35 (52%)*	77 (92%)	21 (27%)
MCV	65 (74%)*	3 (5%)	78 (93%)	4 (5%)
Erythrocytes	58 (66%)	37 (64%)	59 (70%)	30 (51%)
Leukocytes	71 (81%)*	45 (63%)*	80 (95%)	36 (45%)
Leukocyte differentiation	35 (40%)	24 (69%)	37 (44%)	23 (62%)
Thrombocytes	68 (77%)*	6 (9%)	77 (92%)	6 (8%)
TSH	37 (42%)*	8 (22%)	54 (64%)	17 (32%)
AF	56 (64%)*	12 (21%)	66 (79%)	14 (21%)
ALAT	54 (61%)	2 (4%)	62 (74%)	2 (3%)
ASAT	44 (50%)*	5 (11%)	56 (67%)	3 (5%)
GGT	46 (52%)*	10 (22%)	59 (70%)	18 (31%)
LD	36 (41%)	18 (50%)	45 (54%)	17 (38%)
Vitamin B12	27 (31%)*	2 (7%)	42 (50%)	3 (7%)
Folic Acid	25 (28%)*	3 (12%)	38 (45%)	10 (26%)
Creatine	71 (81%)*	20 (28%)	83 (99%)	30 (36%)
Total protein	8 (9%)	2 (25%)	4 (5%)	0 (0%)
CRP*	68 (77%)*	41 (60%)	80 (95%)	48 (60%)

Data are shown as *n* (%). *Statistically significant difference (*p* < 0.05) between nonoperative and operative groups

*CRP was is not included as standard diagnostic test in both guidelines but was added to the table because of its frequency in testing and possible clinical significance

*AF* alkaline phosphatase, *ALAT* alanine transaminase, *ASAT* aspartate transaminase, *CGA* comprehensive geriatric assessment, *CRP* C-reactive protein, *eGFR* estimated glomerular filtration rate, *ESR* erythrocyte sedimentation rate, *GGT* Gamma-glutamyltransferase, *LD* lactate dehydrogenase, *MCV* mean corpuscular volume, *TSH* thyroid stimulating hormone

With regards to consequences for abnormal test results found during laboratory diagnostics (Table 6), for most abnormal test results, no additional diagnostics or interventions were reported. In the nonoperative group, no intervention or additional diagnostics rate was higher for all laboratory diagnostic subgroups, but a significant difference was only found for the hematological (55/64 versus 42/62, *p* = 0.015) and vitamin status groups (8/9 versus 8/21, *p* = 0.011). Abnormal test results in vitamins (1/9 versus 13/21, *p* = 0.011) and inflammatory markers (3/42 versus 12/51, *p* = 0.046) both showed an increased rate of pharmacological intervention in the operative group. Abnormal tests in kidney function were associated with an increased rate of additional diagnostics in the operative group (1/43 versus 11/56, *p* = 0.036).

**Table 6:** Consequences of abnormal diagnostic test results found during laboratory diagnostics or microbiological testing

	Nonoperative				Operative			
	No diagnostics or intervention	Additional diagnostics	Pharmacological^a^ intervention	Invasive intervention	No diagnostics or intervention	Additional diagnostics	Pharmacological^a^ intervention	Invasive intervention
*Laboratory diagnostics*								
Hematological	55 (83%)*	9	4	0	42 (68%)	13	10	0
Electrolytes	21 (84%)	4	2	0	21 (66%)	7	8	0
Nutritional status	35 (95%)	2	0	0	40 (87%)	2	4	0
Vitamin status	8 (89%)*	0	1*	0	8 (38%)	0	13	0
Inflammatory markers	34 (81%)	6	3*	0	33 (65%)	14	12	0
Kidney function	40 (91%)	2*	2	0	47 (84%)	11	4	0
Liver and pancreatic function	36 (97%)	1	0	0	35 (95%)	2	0	0
Cardiac function	1 (33%)	1	1	0	1 (100%)	0	0	0
Thyroid function	9 (91%)	0	1	0	18 (100%)	0	0	0
Coagulation	8 (44%)	8	10	0	4 (21%)	14	14	0
Arterial blood gas	0 (0%)	2	2	0	1 (100%)	0	0	0
Urine	4 (67%)	0	2	0	6 (33%)	4	9	0
*Microbiological diagnostics*								
Blood culture	2 (100%)	0	0	0	0 (0%)	0	1	0
Urine culture	5 (100%)	0	0	0	5 (50%)	0	5	0

Data are shown as *n* (%) of total number of abnormal diagnostic tests per subcategory. *Statistically significant difference (*p* < 0.05) between nonoperative and operative groups. A single patient could receive a combination of additional diagnostics and/or pharmacological- and/or invasive interventions

^a^Not being analgesics

### Laboratory diagnostics in guidelines

Adherence to the (Dutch) hip fracture guideline was investigated for the operative group. Hemoglobin was tested in all 84 patients, 82 (97.6%) patients received at least a sodium and potassium test, albumin was tested in 69 (82.1%) patients, kidney function was tested in 83 (98.8%) patients, and glucose was tested in 80 (95.2%) patients (also shown in Table 5).

In addition to this, in accordance with the Dutch guideline for comprehensive geriatric assessment: In a total of 84 operatively treated patients, in 27 (32%) patients ESR was tested, in 54 (64%) thyroid function was tested, in 68 (81%) at least one of the liver enzymes was tested, in 42 (50%) vitamin B12 was tested, in 38 (45%) folic acid was tested, and in four (5%) total serum protein was tested. A complete blood count was also advised. In addition to hemoglobin in all patients, for 77 (92%) patients hematocrit, for 78 (93%) mean corpuscular volume (93%), erythrocyte count 59 (70%), thrombocyte count 77 (92%), and leukocyte count 80 (95%) was determined, in the operative group. The number and proportion of performed diagnostics in the nonoperative group was generally lower (OR 1, Table S9).

### Delay and change of intervention

Surgery was delayed for > 24 h in 31/84 (36.9%) patients (Table 7). This was most commonly due to logistical reasons (*e.g.*, admission in the early morning, no available timeslot in the surgical program, not possible to contact family in time). In 10/84 (11.0%) patients, new findings (*e.g.*, pneumonia, irregular antibodies, malignancy screening, initial nonoperative treatment changed to operative after increased fracture instability) during diagnostic testing were cited as a factor in delayed surgery (Table 7). In 5/172 (3.0%) patients, new findings were mentioned as one of the arguments for the decision to switch from operative treatment to nonoperative treatment. Specific arguments mentioned were a newly found severe aortic valve dysfunction (cardiac echography), increasing renal and cardiological dysfunction (laboratory diagnostics), liver metastasis (abdominal echography), and/or pulmonary infection/malignancy (thoracic X-ray). In all cases, these were only mentioned in a list of other arguments, such as low quality of life, worsening dementia, low mobility pre-trauma, and weariness of life. Changes from nonoperative to operative treatment in 2/172 (1%) patients were all due to increasing fracture displacement on an additional (secondary) X-ray or CT-scan because of increasing unmanageable pain after initial nonoperative management.

**Table 7:** Reasons for postponement of surgery (> 24 h) and change of operative of nonoperative treatment (partly) due to abnormal diagnostic test results

	Operative (*n* = 84)
Delayed surgery	31 (37%)
*Reason delay*^a^	
New finding	10 (32%)
Known finding	2 (6%)
Coagulation	11 (13%)
Logistical	20 (65%)
Change of treatment due to abnormal diagnostic test results	Total population (*n* = 172)
Operative to nonoperative^b^	5 (3%)
Nonoperative to operative^c^	2 (1%)
Unknown reason	3 (2%)
New abnormal diagnostic tests not mentioned in treatment decision	162 (96%)

Data are shown as *n* (%)

^a^A combination of multiple reasons was possible

^b^Specific test results: Severe aortic valve dysfunction (cardiac echography), increasing renal and cardiological dysfunction (laboratory diagnostics), liver metastasis (abdominal echography), and/or pulmonary infection/malignancy (thoracic X-ray)

^c^Increasing fracture displacement on an additional (secondary) X-ray or CT-scan

## Discussion

This study evaluated (geriatric and preoperative) diagnostic testing in frail institutionalized patients with a hip fracture. The results showed that both a large variety and number of diagnostics were performed in this population, after presentation to the Emergency Department, both in the operative and nonoperative group. Operatively treated patients were tested more extensively in both radiological and laboratory diagnostics and the majority of tests (> 90%) in both groups was requested at or by the Emergency Department. While many abnormal diagnostic tests did not result in (pharmacological) interventions or additional diagnostics, abnormal diagnostic tests were mentioned as a reason for surgery delay and as an argument in the decision between operative and nonoperative hip fracture care in several cases.

Both the operative and nonoperative groups showed large numbers of diagnostics. The rates of radiological diagnostics (not being pelvic X-rays) showed no differences between groups, except for the rate of cardiac ultrasound, which was performed more often in the operative group. Cardiac ultrasounds are often performed in the preoperative work-up in patients with known or newly found signs of heart failure, abnormal ECGs, or cardiac murmurs during physical examination and would thus, logically, be performed more often in operative patients [18, 19]. The number of (preoperative) chest X-rays was relatively high (67% nonoperative and 80% operative) and they were often used without clinical indication, while recent literature and guidelines recommend only indicated use of chest X-rays [14, 18–20]. This study also showed that while abnormal results were found in many cases, only in a small group of patients (4/41 nonoperative and 5/32 operative patients) abnormal findings on chest X-rays were an argument for (pharmacological) intervention. This is in line with results found in previous research [14, 20].

Rates of laboratory and microbiological diagnostics were even higher, with 100% of operative patients and 82% of nonoperative patients undergoing blood tests. The significant differences between groups in performed diagnostics can be primarily explained by a group of 17 conservative patients in which nonoperative care was established before other diagnostics than pelvic X-rays were performed and were subsequently discharged without additional diagnostics.

In the patients who did receive laboratory diagnostics, this study found no significant differences in abnormal test results found per subgroup of laboratory diagnostics except for abnormalities in hematological diagnostics. In this subcategory, nonoperatively treated patients showed more abnormal test results than operatively treated patients. This is probably due to a worse (pre-trauma) condition and related to their choice for nonoperative care for their hip fracture. While there are differences between operative and nonoperative patients in the amount of diagnostic testing, these differences are remarkably small. As most tests were requested at or by the Emergency Department, they were conducted prior to or during the treatment decision. The treatment group might, therefore, have a limited effect on the number of requested tests.

This is the first study on the consequences of diagnostic testing in frail institutionalized older adults with hip fractures and to analyze this in both operative and nonoperative patients. Many previous prognostic studies have studied the influence of abnormal diagnostic tests in laboratory or radiological diagnostics on mortality after hip fracture surgery [21–24]. They concluded that, despite the high predictive value of potential biomarkers, advanced age, minimal pre-trauma mobility and history of ischemic heart disease are the most important predictors for (in-hospital) mortality after a hip fracture [23–25]. Prediction of in-hospital mortality may be an essential argument in the decision between operative and nonoperative care for patients with a hip fracture. As this was a retrospective analysis, the added value of specific tests in the treatment decision could not be studied.

(Trauma-)surgical guidelines on preoperative screening in older adults with a hip fracture advise restricting diagnostic testing and treatment to only acute correctable comorbidities or indicated diagnostics [7, 19, 26]. According to surgical guidelines, operations should not be delayed due to abnormalities in the diagnostic process unless there is a severe condition preventing surgery. Because of this, the Dutch guideline advises only a limited blood analysis [7]. In the operative subgroup only hemoglobin was tested in 100% of the patients, with the other tests (except for albumin) performed in 95% of the cases. No previous study evaluated adherence to this part of the guideline. The Dutch guideline also advises multidisciplinary care involving, ideally, a consulting geriatrician. A documented geriatric consultation was achieved in 67% of nonoperative patients and 92% of operative patients (in the period until respectively discharge or operation). In operative patients, this percentage could have increased further after the operation. However, this warrants attention in the nonoperative group as a geriatric opinion combined with the surgeon’s view could be essential in deciding between operative and palliative hip fracture care. In both cases a geriatrician could provide advice and treatment for further recovery and rehabilitation or identify barriers and problems for palliative care.

Many of the laboratory diagnostics performed in this study, such as liver/pancreas function, vitamin status, or thyroid function, do not fit in the limited minimal surgical assessment. Many of those do, however, fit in the comprehensive geriatric assessment (CGA). This assessment is also advised by these same guidelines and plays a vital role in optimizing patients post-operatively for rehabilitation or prevention of complications and fracture recurrence [7, 17, 26–29]. This guideline was met in lower percentages than the surgical guideline. However, additional tests can be expected to have been performed after surgery. It is important to note that a complete CGA was not common practice in all included hospitals and some items, like vitamin status, would play a marginal role in preoperative assessment.

As most test are requested at arrival, due to often standardized ER protocols, one could argue that some of these tests could be postponed until after surgery and could thus be omitted for patients opting for nonoperative/palliative care. These tests are associated with additional costs (*i.e.*, €47 for a thoracic X-ray and €46 for a vitamin status) and several seem to have limited direct value for both clinician and patient [30]. On the other hand, grouping (blood) tests or applying standardized diagnostic protocols for whole patient groups can increase organizational efficiency and reduce the number of different diagnostic intervals during a patients hospital stay. This creates a dilemma: on the one hand more extensive (standardized) diagnostics for an informed (nonoperative) treatment decision, and on the other hand doing only what is necessary to reduce the additional patient burden and associated costs. Either approach’s effect has yet to be studied but could be included in a future (cost-effectiveness) analysis. While diagnostic tests were mentioned as one of the reasons for change in treatment decisions between operative and nonoperative care, they were always mentioned in conjunction with other arguments about patients pre-existing health status or treatment wishes. Most tests were requested shortly after arrival at the ER, probably prior to an extensive treatment decision, however, the exact timing of a treatment decision is hard to establish and the role of specific (normal or abnormal) test results is often not described. Previous research on the shared decision making process identified co-morbidities as an important argument for palliative care after hip fracture but did not identify specific test results [31]. The weight of abnormal diagnostic test results, as an argument, is unclear and would probably vary between patients.

This study was a secondary retrospective analysis, and thus results should be interpreted with care. In many cases, the clinical thought process remains unclear as reasons for performed tests were often not documented and consequences of abnormal diagnostic tests may have been considered but not performed or documented. A clear clinical indication is often absent when retrospectively accessing a medical file and clinical advice may have been given by other specialists but not officially documented as consultation. Additionally, this study only analyzed test outcomes as described in the medical files. Test results (*e.g.*, X-rays or electrocardiograms) were not interpreted by the researchers. Abnormal findings could have been missed, especially if found irrelevant at the time by the initial observer. Findings corresponding with chronic conditions such as arthrosis or cardiomegaly may not have been reported when a test’s indication was to find acute diseases (*e.g.*, hip fracture or pneumonia) and new abnormal findings might be a result of existing disease not registered in the hospital of admission. As a result of these limitations, the results of this study should primarily be used as a basis for further prospective diagnostic and medical decision-making research. As nonoperative, palliative treatment for hip fractures in the frail older adults is becoming a viable and more accepted treatment option, it becomes crucial to define what additional diagnostics and consultations are necessary (or redundant) in deciding between operative and nonoperative care.

## Conclusion

When frail older adults are admitted with a hip fracture, an extensive diagnostic process is started including a large number and variety of laboratory and radiological diagnostics. Operatively treated patients are tested more extensively than nonoperatively treated patients. Abnormal test results in laboratory diagnostics are found for almost all patients, however, many abnormal findings seem to have no clinical consequences. In few cases, diagnostic tests seem to influence treatment decisions and in a proportion of patients diagnostics are an argument for delay in the time till operation. However, the exact importance of diagnostic testing in these decisions remains unclear. As nonoperative hip fracture care is becoming a more viable palliative treatment option, prospective research is required to evaluate the timing and added value of the separate elements of preoperative diagnostic testing and geriatric assessment in older adults with hip fractures.

### Supplementary Information

Below is the link to the electronic supplementary material.

Supplementary file1 (DOCX 42 KB)

Supplementary file2 (DOCX 17 KB)

## Data Availability

All data used are presented in the manuscripts tables or Online resource 1. The corresponding author can be contacted for additional requests or questions regarding the data or analysis.
