# Supplementary material for: The value of preoperative diagnostic testing and geriatric assessment in frail institutionalized elderly with a hip fracture; a secondary analysis of the FRAIL-HIP study
Source: Eur Geriatr Med. 2024 Feb 28;15(3):753–63. doi: 10.1007/s41999-024-00945-8 (PMC11329590; doi:10.1007/s41999-024-00945-8)
Supplement: Supplementary file 1 — Supplementary file1 (DOCX 42 KB) [file 41999_2024_945_MOESM1_ESM.docx]

**Online Resource 1: Supplemental tables**

**Table S1: Findings reported during physical examination at admission**

|  | **Nonoperative** | | **Operative** | |
| --- | --- | --- | --- | --- |
|  | Total patients with measurement | n | Total patients with measurement | n |
| **Cardiovascular abnormalities** | 70 |  | 67 |  |
| Cardiac souffle |  | 20 |  | 16 |
| Arrhythmia |  | 4 |  | 5 |
| No abnormalities |  | 47 |  | 47 |
| **Pulmonary abnormalities** | 71 |  | 73 |  |
| Crepitations |  | 7 |  | 5 |
| Rhonchi |  | 1 |  | 5 |
| Decreased auscultation volume |  | 1 |  | 0 |
| No abnormalities |  | 62 |  | 64 |
| **Abdominal abnormalities** | 56 |  | 60 |  |
| No abnormalities |  | 56 |  | 60 |
| **Neurological abnormalities** | 45 |  | 55 |  |
| Reduced consciousness |  | 3 |  | 1 |
| Pin-point pupils |  | 2 |  | 1 |
| None |  | 40 |  | 55 |

**Table S2: Screening versus indicated reason for radiological diagnostics and electrocardiograms**

|  | **Nonoperative**  **(n=88)** | | **Operative**  **(n=84)** | |
| --- | --- | --- | --- | --- |
|  | Screening  n | Indicated  n | Screening  n | Indicated  n |
| **X-rays** |  |  |  |  |
| Pelvis | 0 | 88 | 0 | 84 |
| Thorax | 57 | 2 | 65 | 2 |
| Shoulder/clavicle | N.A. | N.A. | 0 | 3 |
| Arm/wrist | 0 | 6 | 0 | 6 |
| Hand | N.A. | N.A. | 1 | 0 |
| Upper leg | 0 | 2 | N.A. | N.A. |
| Knee | 1 | 2 | 0 | 5 |
| Lower leg | 0 | 1 | 0 | 1 |
| **CT-scans** |  |  |  |  |
| Pelvis | 0 | 2 | 0 | 6 |
| Thorax | 0 | 1 | 0 | 1 |
| Cervical spine | 0 | 4 | 0 | 6 |
| Head | 0 | 8 | 1 | 7 |
| Face | 0 | 1 | N.A. | N.A. |
| **Ultrasound** |  |  |  |  |
| Cardiac | 0 | 4 | 3 | 11 |
| Abdominal | 1 | 0 | N.A. | N.A. |
| **Electrocardiograms** | 46 | 1 | 68 | 1 |

N.A., not applicable

**Table S3: Screening versus indicated reason for laboratory and microbiological diagnostics**

|  | **Nonoperative**  **(n=72)** | | **Operative**  **(n=84)** | |
| --- | --- | --- | --- | --- |
|  | Screening  n | Indicated  n | Screening  n | Indicated  n |
| **Laboratory testing** |  |  |  |  |
| Hematological lab | 72 | 0 | 84 | 0 |
| Electrolytes and vitamins | 72 | 1 | 82 | 3 |
| Nutritional status | 67 | 0 | 81 | 0 |
| Inflammatory markers | 69 | 0 | 80 | 0 |
| Kidney function | 71 | 0 | 83 | 0 |
| Liver and pancreatic function | 61 | 0 | 68 | 0 |
| Cardiac function | 1 | 1 | 1 | 0 |
| Thyroid function | 37 | 0 | 52 | 2 |
| Coagulation | 58 | 4 | 77 | 5 |
| Arterial blood gas | 2 | 1 | 1 | 0 |
| Urine sediment | 22 | 0 | 43 | 3 |
| **Microbiological testing** |  |  |  |  |
| Blood culture | 1 | 2 | 1 | 5 |
| Urine culture | 5 | 4 | 7 | 8 |
| MRSA culture | 1 | 0 | 1 | 1 |
| Viral PCR | 0 | 0 | 0 | 1 |

**Table S4: Requesting departments for radiological diagnostics and electrocardiograms**

|  | **Nonoperative**  n=88 | **Operative**  n=84 |
| --- | --- | --- |
| **X-Pelvis** |  |  |
| Emergency department | 81 | 71 |
| Surgery | 5 | 9 |
| Orthopedics | 2 | 4 |
| **X-Thorax** |  |  |
| Emergency department | 54 | 54 |
| Surgery | 2 | 8 |
| Orthopedics | 2 | 4 |
| Geriatrics | 0 | 1 |
| ICU | 1 | 0 |
| **X-Shoulder** |  |  |
| Emergency department | 0 | 3 |
| **X-Arm/wrist** |  |  |
| Emergency department | 6 | 5 |
| Surgery | 0 | 1 |
| **X-Hand** |  |  |
| Emergency department | 0 | 1 |
| **X-Upper leg** |  |  |
| Emergency department | 2 | 0 |
| **X-Knee** |  |  |
| Emergency department | 1 | 3 |
| Surgery | 2 | 1 |
| Orthopedics | 0 | 1 |
| **X-Lower leg** |  |  |
| Emergency department | 1 | 1 |
| **CT-Pelvis** |  |  |
| Emergency department | 2 | 3 |
| Surgery | 0 | 2 |
| Radiology | 0 | 1 |
| **CT-Thorax** |  |  |
| Emergency department | 0 | 1 |
| Pulmonary medicine | 1 | 0 |
| **CT-Cervical spine** |  |  |
| Emergency department | 3 | 5 |
| Surgery | 1 | 0 |
| Neurology | 0 | 1 |
| **CT-Head** |  |  |
| Emergency department | 5 | 6 |
| Surgery | 1 | 0 |
| Neurology | 2 | 1 |
| Geriatrics | 0 | 1 |
| **CT-Face** |  |  |
| Emergency department | 0 | 1 |
| **Cardiac ultra-sound** |  |  |
| Surgery | 0 | 3 |
| Orthopedics | 0 | 2 |
| Geriatrics | 0 | 1 |
| Cardiology | 4 | 7 |
| Anesthesiology | 0 | 1 |
| **Abdominal ultrasound** |  |  |
| Internal medicine | 1 | 0 |
| **Electrocardiogram** |  |  |
| Emergency medicine | 40 | 55 |
| Surgery | 4 | 6 |
| Orthopedics | 0 | 4 |
| Geriatrics | 1 | 1 |
| Cardiology | 2 | 3 |

ICU, intensive care unit

**Table S5: Requesting departments for laboratory diagnostics**

|  | **Nonoperative**  (n=72) | **Operative**  (n=84) |
| --- | --- | --- |
| **Hematological lab** |  |  |
| Emergency medicine | 71 | 79 |
| Surgery | 5 | 11 |
| Orthopedics | 4 | 5 |
| Geriatrics | 1 | 1 |
| **Electrolytes and vitamins** |  |  |
| Emergency medicine | 71 | 77 |
| Surgery | 5 | 11 |
| Orthopedics | 4 | 5 |
| Geriatrics | 4 | 11 |
| ICU | 1 | 0 |
| **Nutritional status** |  |  |
| Emergency medicine | 66 | 77 |
| Surgery | 4 | 10 |
| Orthopedics | 4 | 5 |
| Geriatrics | 2 | 3 |
| **Inflammatory markers** |  |  |
| Emergency medicine | 68 | 76 |
| Surgery | 4 | 10 |
| Orthopedics | 4 | 5 |
| Geriatrics | 1 | 0 |
| **Kidney function** |  |  |
| Emergency medicine | 70 | 78 |
| Surgery | 5 | 11 |
| Orthopedics | 4 | 5 |
| Geriatrics | 1 | 2 |
| **Liver and pancreatic function** |  |  |
| Emergency medicine | 59 | 63 |
| Surgery | 4 | 9 |
| Orthopedics | 4 | 5 |
| Geriatrics | 1 | 3 |
| ICU | 1 | 0 |
| **Cardiac function** |  |  |
| Cardiology | 2 | 1 |
| **Thyroid function** |  |  |
| Emergency medicine | 35 | 44 |
| Surgery | 3 | 6 |
| Orthopedics | 0 | 2 |
| Geriatrics | 1 | 6 |
| **Coagulation** |  |  |
| Emergency medicine | 60 | 77 |
| Surgery | 5 | 10 |
| Orthopedics | 4 | 5 |
| Geriatrics | 1 | 0 |
| **Arterial blood gas** |  |  |
| Emergency medicine | 2 | 0 |
| Geriatrics | 0 | 1 |
| ICU | 1 | 0 |
| **Urine sediment** |  |  |
| Emergency medicine | 20 | 41 |
| Surgery | 2 | 7 |
| Orthopedics | 3 | 6 |
| Geriatrics | 1 | 2 |
| ICU | 1 | 0 |
| **D-dimer** |  |  |
| Emergency medicine | 0 | 1 |

**Table S6: Consequences of (non-surgical) clinical consultations**

|  | **Nonoperative**  **(n=88)** | | | | | **Operative**  **(n=84)** | | | | |
| --- | --- | --- | --- | --- | --- | --- | --- | --- | --- | --- |
| **Consultation** | n | No diagnostics or intervention | Additional diagnostics | Pharmacological^a^  intervention | Invasive intervention | n | No diagnostics or intervention | Additional diagnostics | Pharmacological^a^  intervention | Invasive intervention |
| Geriatrics | 59 | 39 (66%) | 7 (12%) | 18 (31%) | 0 | 77 | 19 (25%) | 28 (36%) | 50 (65%) | 0 |
| Cardiology | 8 | 2 (25%) | 6 (75%) | 1 (13%) | 0 | 22 | 7 (32%) | 13 (59%) | 3 (14%) | 0 |
| Neurology | 2 | 1 | 1 | 0 | 0 | 4 | 2 | 1 | 1 | 0 |
| Internal medicine | 1 | 0 | 1 | 0 | 0 | 3 | 0 | 1 | 2 | 0 |
| Pulmonary medicine | 2 | 0 | 2 | 2 | 0 | 1 | 1 | 0 | 0 | 0 |
| Anesthesia | 2 | 2 | 0 | 0 | 0 | 0 | - | - | - | - |
| Other^b^ | 1 | 1 | 0 | 0 | 0 | 4 | 1 | 2 | 0 | 1^c^ |

Data are shown as n (%).

^a^ Not being only analgesics

^b^ Other including: Psychiatry, Microbiology, ophthalmology, urology

^c^ The single invasive intervention after consultation was a cystoscopic catheter placement by the urology department

**Table S7: Abnormal findings found during radiological diagnostics and electrocardiograms**

|  | **Nonoperative**  **(n=88)** | | **Operative**  **(n=84)** | |
| --- | --- | --- | --- | --- |
|  | New finding | Known finding | New finding | Known finding |
| **X-Pelvis** |  |  |  |  |
| Fracture | 87 | 0 | 84 | 0 |
| Arthrosis | 14 | 3 | 14 | 5 |
| Other^a^ | 0 | N.A. | 2 | 0 |
| No abnormal findings | 1 | N.A. | 0 | N.A. |
| **X-thorax** |  |  |  |  |
| Fracture | 2 | 1 | 5 | 0 |
| Cardiomegaly | 18 | 9 | 15 | 10 |
| Consolidation | 8 | 1 | 8 | 0 |
| Nodule/mass | 4 | 0 | 1 | 0 |
| Hilar enlargement | 4 | 0 | 2 | 0 |
| Atelectasis | 0 | N.A. | 3 | 0 |
| Fibrosis/interstitial pattern | 8 | 1 | 5 | 1 |
| Pulmonary edema | 3 | 0 | 3 | 2 |
| Pleural effusion | 7 | 1 | 4 | 0 |
| Other^b^ | 4 | 1 | 3 | 3 |
| No abnormal findings | 18 | N.A. | 35 | N.A. |
| **X-shoulder** |  |  |  |  |
| Fracture | N.A. | N.A. | 2 | 0 |
| No abnormal findings | N.A. | N.A. | 1 | 0 |
| **X-arm** |  |  |  |  |
| Fracture | 5 | 0 | 4 | 0 |
| No abnormal findings | 1 | N.A. | 2 | N.A. |
| **X-hand** |  |  |  |  |
| Arthrosis | N.A. | N.A. | 1 | 1 |
| **X-upper leg** |  |  |  |  |
| No abnormal findings | 0 | N.A. | N.A. | N.A. |
| **X-knee** |  |  |  |  |
| Arthrosis | 0 | N.A. | 3 | 2 |
| No abnormal findings | 3 | N.A. | 2 | N.A. |
| **X-lower leg** |  |  |  |  |
| Fracture | 1 | 0 | 0 | N.A. |
| No abnormal findings | 0 | N.A. | 1 | N.A. |
| **CT-pelvis** |  |  |  |  |
| Fracture | 2 | 0 | 6 | 0 |
| Arthrosis | 0 | N.A. | 2 | 0 |
| **CT-thorax** |  |  |  |  |
| Aortic aneurysm | 1 | 0 | 0 | N.A. |
| Atelectasis | 1 | 0 | 0 | N.A. |
| Pulmonary embolism | 0 | N.A. | 1 | 0 |
| Diaphragm herniation | 0 | N.A. | 1 | 0 |
| **CT-cervical spine** |  |  |  |  |
| Arthrosis | 3 | 1 | 4 | 0 |
| Thyroid nodule | 1 | 0 | 0 | N.A. |
| No abnormal findings | 1 | N.A. | 2 | N.A. |
| **CT-head** |  |  |  |  |
| Cerebral atrophy | 5 | 4 | 5 | 3 |
| Cerebral infarction | 1 | 0 | 2 | 2 |
| White matter lesion | 1 | 1 | 1 | 1 |
| No abnormal findings | 3 | N.A. | 2 | N.A. |
| **CT-face** |  |  |  |  |
| No abnormal findings | 1 | N.A. | N.A. | N.A. |
| **Cardiac ultrasound** |  |  |  |  |
| Valve dysfunction | 4 | 1 | 8 | 1 |
| Left dysfunction | 0 | N.A. | 2 | 2 |
| Pulmonary hypertension | 0 | N.A. | 2 | 0 |
| No abnormal findings | 0 | N.A. | 4 | N.A. |
| **Abdominal ultrasound** |  |  |  |  |
| Liver metastasis | 1 | 0 | N.A. | N.A. |
| **Electrocardiograms** |  |  |  |  |
| Conduction disorder^c^ | 31 | 21 | 39 | 24 |
| STEMI | 1 | 0 | 0 | N.A. |
| Non-STEMI | 0 | N.A. | 1 | 0 |
| Heart failure | 0 | N.A. | 1 | 0 |
| No abnormal findings | 15 | N.A. | 28 | N.A. |

^a^Includes a myoma and myositis ossificans

^b^Includes one trachea deviation, two diaphragm herniations, two aortic enlargements, one scoliosis and one shoulder arthrosis

^c^Including atrium fibrillation

N.A., not applicable

**Table S8: Consequences of abnormalities found during radiological diagnostics and electrocardiograms**

|  | **Nonoperative**  **(n=88)** | | | | | **Operative**  **(n=84)** | | | | |
| --- | --- | --- | --- | --- | --- | --- | --- | --- | --- | --- |
|  | n | No diagnostics or intervention | Additional diagnostics | Pharmacological^a^  intervention | Invasive intervention | n | No diagnostics or intervention | Additional diagnostics | Pharmacological^a^  intervention | Invasive intervention |
| **X-rays** |  |  |  |  |  |  |  |  |  |  |
| Pelvis | 87 | 85 | 1 | 1 | 0 | 84 | 0 | 5 | 0 | 82 |
| Thorax | 41 | 37 | 1 | 4 | 0 | 32 | 27 | 3 | 5 | 0 |
| Shoulder/clavicle | 0 | N.A. | N.A. | N.A. | N.A. | 2 | 2 | 0 | 0 | 0 |
| Arm/wrist | 5 | 4 | 0 | 0 | 1 | 4 | 2 | 0 | 0 | 2 |
| Hand | 0 | N.A. | N.A. | N.A. | N.A. | 1 | 1 | 0 | 0 | 0 |
| Upper leg | 0 | N.A. | N.A. | N.A. | N.A. | 0 | N.A. | N.A. | N.A. | N.A. |
| Knee | 0 | N.A. | N.A. | N.A. | N.A. | 3 | 3 | 0 | 0 | 0 |
| Lower leg | 1 | 1 | 0 | 0 | 0 | 0 | N.A. | N.A. | N.A. | N.A. |
| **CT-scan** |  |  |  |  |  |  |  |  |  |  |
| Pelvis | 2 | 2 | 0 | 0 | 0 | 6 | 1 | 0 | 0 | 5 |
| Thorax | 1 | 1 | 0 | 0 | 0 | 1 | 0 | 0 | 1 | 0 |
| Cervical spine | 3 | 3 | 0 | 0 | 0 | 4 | 4 | 0 | 0 | 0 |
| Head | 5 | 5 | 0 | 0 | 0 | 6 | 6 | 0 | 0 | 0 |
| Face | 0 | N.A. | N.A. | N.A. | N.A. | 0 | N.A. | N.A. | N.A. | N.A. |
| **Ultrasound** |  |  |  |  |  |  |  |  |  |  |
| Cardiac | 4 | 4 | 0 | 0 | 0 | 10 | 10 | 0 | 0 | 0 |
| Abdomen | 1 | 1 | 0 | 0 | 0 | 0 | N.A. | N.A. | N.A. | N.A. |
| **ECGs** | 32 | 30 | 1 | 0 | 0 | 41 | 38 | 2 | 1 | 0 |

^a^Not being analgesics

N.A., not applicable

**Table S9: Specific laboratory diagnostic outcomes for nonoperative and operative patients**

|  | **Nonoperative**  **n=88** | | **Operative**  **n=84** | |  |
| --- | --- | --- | --- | --- | --- |
|  | n | Mean (SD)/  Median (P25-P75) | n | Mean (SD)/  Median (P25-P75) | P-value |
| **Hematological** | **72 (82%)** | **-** | **84 (100%)** |  |  |
| Hemoglobin (mmol/L) | 72 | 7.4 (SD 1.1) | 84 | 7.8 (SD 0.87) | **0.009** |
| Hematocrit (mmol/L) | 68 | 0.36 (SD 0.052) | 77 | 0.38 (SD 0.040) | **0.016** |
| MCV (fL) | 65 | 92 (89-96) | 78 | 93 (89-96) | 0.765 |
| Erythrocytes (10^12^/L) | 58 | 4.0 (SD 0.63) | 59 | 4.0 (SD 0.53) | 0.459 |
| Thrombocytes (10^9^/L) | 68 | 244 (204-296) | 77 | 239 (199-293) | 0.387 |
| Leukocytes (10^9^/L) | 71 | 10.8 (9.1-13.3) | 80 | 9.9 (7.7-13.0) | 0.066 |
| Leukocyte differentiation | 35 | - | 37 | - | N.A. |
| **Electrolytes** | **72 (82%)** |  | **82 (98%)** |  |  |
| Sodium (mmol/L) | 72 | 139 (137-141) | 82 | 139 (137-141) | 0.561 |
| Chlorine (mmol/L) | 17 | 104 (SD 5.1) | 15 | 105 (SD 2.9) | 0.289 |
| Potassium (mmol/L) | 72 | 4.1 (SD 0.50) | 81 | 4.3 (SD 0.50) | 0.11 |
| Calcium (mmol/L) | 58 | 2.29 (SD 0.14) | 68 | 2.30 (SD 0.12) | 0.768 |
| Magnesium (mmol/L) | 4 | 0.82 (SD 0.10) | 7 | 0.88 (SD 0.090) | 0.274 |
| Phosphate (mmol/L) | 11 | 0.99 (SD 0.23) | 10 | 1.06 (SD 0.19) | 0.448 |
| Bicarbonate (mmol/L) | 2 | 20.1 (N.A) | 3 | 25.0 (N.A.) | 0.248 |
| Anion gap (meq/L) | 1 | 10 (N.A.) | 2 | 9.5 (N.A.) | 1 |
| Iron (mmol/L) | 5 | 9.3 (SD 4.9) | 13 | 6.5 (SD 3.2) | 0.173 |
| Ferritin (ug/L) | 7 | 128 (SD 101) | 15 | 190 (SD 142) | 0.316 |
| Transferrin (g/L) | 2 | 2.1 (N.A.) | 12 | 2.1 (2.0-2.5) | 0.703 |
| Iron saturation (%) | 0 | N.A. | 2 | 16 (N.A.) | N.A. |
| **Nutritional status** | **67 (76%)** |  | **81 (96%)** |  |  |
| Glucose (mmol/L) | 66 | 7.0 (6.3-7.7) | 80 | 7.4 (6.4-8.6) | 0.060 |
| Total protein (g/L) | 8 | 65 (SD 5.3) | 4 | 74 (SD 4.2) | **0.015** |
| Albumin (g/L) | 58 | 34 (29-36) | 69 | 35 (32-39) | **0.023** |
| **Vitamin status** |  |  |  |  |  |
| Vitamin D (nmol/L) | 60 | 74 (SD 30) | 46 | 76 (SD 26) | 0.765 |
| Vitamin B1 (nmol/L) | 3 | 141 (SD 16) | 10 | 103 (SD 27) | **0.041** |
| Vitamin B6 (nmol/L) | 3 | 68 (SD 43) | 8 | 41 (SD 19) | 0.175 |
| Folic acid (nmol/L) | 25 | 18 (SD 13) | 38 | 17 (SD 13) | 0.603 |
| Vitamin B12 (pmol/L) | 27 | 225 (169-437) | 42 | 240 (96-415) | 0.796 |
| **Inflammatory markers** | **69 (78%)** |  | **80 (95%)** |  |  |
| CRP (mg/L) | 68 | 10 (5-36) | 80 | 11 (4.3-32) | 0.998 |
| ESR (mm/hour) | 24 | 23 (12-34) | 27 | 26 (20-44) | 0.138 |
| Venous lactate (mmol/L) | 2 | 2.2 (N.A.) | 0 | N.A. | N.A. |
| **Kidney function** | **71 (81%)** |  | **83 (99%)** |  |  |
| eGFR (mL/min/1,73m^2^) | 71 | 65 (49-78) | 83 | 60 (42-73) | **0.026** |
| Creatine (µmol/L) | 71 | 72 (58-99) | 83 | 84 (65-104) | 0.074 |
| Ureum (mmol/L) | 65 | 7.7 (6.4-9.8) | 78 | 8.5 (6.6-10.6) | 0.236 |
| **Liver/pancreas function** | **61 (69%)** |  | **68 (81%)** |  |  |
| ALAT (U/L) | 54 | 17 (12-23) | 62 | 16 (13-20) | 0.286 |
| ASAT (U/L) | 44 | 21 (18-28) | 56 | 21 (17-25) | 0.377 |
| AF (U/L) | 56 | 86 (76-109) | 66 | 87 (71-101) | 0.442 |
| GGT (U/L) | 46 | 22 (12-43) | 59 | 29 (17-59) | 0.128 |
| LD (U/L) | 36 | 246 (200-297) | 45 | 223 (201-276) | 0.500 |
| CK (U/L) | 29 | 66 (39-109) | 25 | 59 (46-94) | 0.658 |
| Total bilirubin (µmol/L) | 30 | 9.2 (6.1-14) | 36 | 9.0 (6.1-16) | 0.985 |
| Conjugated bilirubin (µmol/L) | 1 | 23 (N.A.) | 4 | 5.3 (3.3-8.4) | 0.157 |
| Amylase (U/L) | 12 | 61 (SD 22) | 5 | 94 (SD 40) | 0.149 |
| Lipase (U/L) | 15 | 25 (11-139) | 15 | 34 (24-48) | 0.852 |
| **Cardiac function** | **2 (2%)** |  | **1 (1%)** |  |  |
| NT-proBNP (pmol/L) | 2 | 5928 (N.A.) | 1 | 5472 (N.A.) | 1 |
| Troponin T (μg/L) | 1 | 0.064 | 0 | N.A. | N.A. |
| **(Para-)Thyroid function** | **37 (42%)** |  | **54 (64%)** |  |  |
| TSH (mU/L) | 37 | 1.6 (0.85-2.8) | 54 | 2.4 (1.5-5.4) | **0.005** |
| T4 (pmol/L) | 11 | 17 (12-20) | 23 | 15 (12-17) | 0.146 |
| PTH (pmol/L) | 2 | 7.1 (N.A.) | 0 | N.A. | N.A. |
| **Coagulation** | **62 (71%)** |  | **81 (96%)** |  |  |
| Type & screen | 59 | - | 80 | - | N.A. |
| INR | 27 | 2.2 (1.0-2.8) | 30 | 1.2 (1.0-2.3) | 0.641 |
| PT (seconds) | 6 | 16 (13-29) | 7 | 15 (12-17) | 0.475 |
| APTT (seconds) | 1 | 43 (N.A.) | 5 | 28 (26-32) | 0.143 |
| **Arterial blood gas** | **3 (3%)** |  | **1 (1%)** |  |  |
| pH | 3 | 7.41 (7.37-7.41) | 1 | 7.32 (N.A.) | 0.180 |
| PO2 (kPa) | 3 | 7.0 (5.0-7.0) | 1 | 3.9 (N.A.) | 0.180 |
| PCO2 (kPa) | 3 | 5.4 (3.2-5.4) | 1 | 5.6 (N.A.) | 0.655 |
| Bicarbonate (mmol/L) | 3 | 25.0 (15.4-25.0) | 1 | 22.0 (N.A.) | 0.655 |
| Base-excess | 2 | 1.2 (N.A.) | 0 | N.A. | N.A. |
| Lactate (mmol/L) | 1 | 8.5 (N.A.) | 0 | N.A. | N.A. |
| **Other** |  |  |  |  |  |
| D-dimer | 0 | N.A. | 1 | 25000 (N.A.) | N.A. |

Data are shown as n (%), mean (SD) or median (P_25_-P_75_).

**Bold numbers** represent numbers and percentages for the whole subgroup of laboratory diagnostics

AF, alkaline phosphatase; ALAT, alanine transaminase; APTT, Activated partial thromboplastin time; ASAT, aspartate transaminase; CK, creatine kinase; CRP, C-reactive protein; eGFR, estimated glomerular filtration rate; fL, femtoliter; ESR, erythrocyte sedimentation rate; GGT, Gamma-glutamyltransferase; INR, international normalized ratio; kPa, kilopascal; LD, lactate dehydrogenase; L, liter; m, meter; MCV, mean corpuscular volume; mg, milligram; mm, millimeter; mmol, millimol; μg, microgram; μmol, micromol; NT-proBNP, N-terminal prohormone of brain natriuretic peptide; PCO_2,_ partial pressure of carbondioxide; pmol, picomol; PO_2_, partial pressure of oxygen; PT, prothrombin time; PTH, parathyroid hormone; P25, 25th percentile; P75, 75th percentile; SD, standard deviation; TSH, thyroid stimulating hormone; T4, thyroxine; U, units

**The value of preoperative diagnostic testing and geriatric assessment in frail institutionalized elderly with a hip fracture; a secondary analysis of the FRAIL-HIP study**

European Geriatric Medicine

Miliaan L. Zeelenberg MD^1^, Dennis Den Hartog MD PhD^1^, Esther. M.M. Van Lieshout PhD MSc^1^; Hugo H. Wijnen MD^2^, Hanna C. Willems MD PhD^3^, Taco Gosens MD PhD^4^, Jeroen Steens MD PhD^5^, Romke Van Balen MD PhD^6^, Rutger G. Zuurmond MD PhD^7^, Sverre A.I. Loggers^1,8^ MD, Pieter Joosse MD PhD^8^, Michael H.J. Verhofstad MD PhD^1^; on behalf of FRAIL-HIP Investigator Group^9^

^1^Trauma Research Unit Department of Surgery, Erasmus MC, University Medical Center Rotterdam, Rotterdam, The Netherlands

^2^Department of Clinical Geriatrics, Rijnstate Hospital, Arnhem, The Netherlands

^3^Department of Internal Medicine and Geriatrics, Amsterdam University Medical Center, Amsterdam, The Netherlands

^4^Department of Orthopedics, Elisabeth Hospital (ETZ), Tilburg, The Netherlands

^5^Department of Surgery, Dijklander Hospital, Hoorn, The Netherlands

^6^Department of Public Public Health and Primary Care, Leiden University Medical Center, Leiden, The Netherlands

^7^Department of Orthopedics, Isala Hospital, Zwolle, The Netherlands

^8^Department of Surgery, Noordwest Ziekenhuisgroep, Alkmaar, The Netherlands

^9^FRAIL-HIP Investigator Group is listed in Online Resource 2

**Corresponding author**

Dr. E.M.M. Van Lieshout

Trauma Research Unit Department of Surgery

Erasmus MC, University Medical Center Rotterdam

P.O. Box 2040

3000 CA Rotterdam

The Netherlands

Phone: +31.10.7031050

Mail: e.vanlieshout@erasmusmc.nl
