# Supplementary material for: The value of preoperative diagnostic testing and geriatric assessment in frail institutionalized elderly with a hip fracture; a secondary analysis of the FRAIL-HIP study
Source: Eur Geriatr Med. 2024 Feb 28;15(3):753–63. doi: 10.1007/s41999-024-00945-8 (PMC11329590; doi:10.1007/s41999-024-00945-8)
Supplement: Supplementary file 2 — Supplementary file2 (DOCX 17 KB) [file 41999_2024_945_MOESM2_ESM.docx]

**Online Resource 2: List of FRAIL-HIP study collaborators**

| **First Name(s)** | **Last name** | **Academic Degree** | **Institution** | **Location** |
| --- | --- | --- | --- | --- |
| Lisanne | Balemans | MD | Flevoziekenhuis | Almere |
| Frank W. | Bloemers | MD PhD | Amsterdam UMC | Amsterdam |
| Janneke | Bos | MD | Catharina Ziekenhuis | Eindhoven |
| Bart J. | Burger | MD PhD | Noordwest Ziekenhuisgroep | Alkmaar |
| Judella O. | Daal | MD | Dijklander Ziekenhuis | Hoorn |
| Annemarieke | De Jonghe | MD PhD | Tergooi | Hilversum |
| Matthea | Dijkshoorn | MD | Ziekenhuis Tjongerschans | Heerenveen |
| Michael J.R. | Edwards | MD PhD | Radboud UMC | Nijmegen |
| Ellen A. | Elbrecht | MD | Deventer Ziekenhuis | Deventer |
| Miriam C. | Faes | MD PhD | Amphia Ziekenhuis | Breda |
| Elvira R. | Flikweert | MD | Deventer Ziekenhuis | Deventer |
| Ellis C. | Folbert | MD PhD | Ziekenhuisgroep Twente | Almelo |
| Robert D.A. | Gaasbeek | MD PhD | Meander MC | Amersfoort |
| Olivia C. | Geraghty | MD PhD | St. Antonius Ziekenhuis | Nieuwegein |
| Taco | Gosens | MD PhD | Elisabeth‐TweeSteden Ziekenhuis | Tilburg |
| J. Carel | Goslings | MD PhD | OLVG | Amsterdam |
| Johannes H. | Hegeman | MD PhD | Ziekenhuisgroep Twente | Almelo |
| Mischa M. | Hindriks | MD | Bernhoven | Uden |
| Micha | Holla | MD PhD | Radboud UMC | Nijmegen |
| André | Janse | MD | Ziekenhuis Gelderse Vallei | Ede |
| Joris A. | Jansen | MD | Alrijne Ziekenhuis | Leiderdorp |
| Simone J.M. | Jong | MD | OLVG | Amsterdam |
| Paul J.C. | Kapitein | MD | Ziekenhuis Gelderse Vallei | Ede |
| Ydo V. | Kleinlugtenbelt | MD PhD | Deventer Ziekenhuis | Deventer |
| Barbara E. | Kreis | MD | Streekziekenhuis Koningin Beatrix | Winterswijk |
| Rover | Krips | MD PhD | Flevoziekenhuis | Almere |
| Koen W.W. | Lansink | MD PhD | Elisabeth‐TweeSteden Ziekenhuis | Tilburg |
| Michiel | Leijnen | MD | Alrijne Ziekenhuis | Leiderdorp |
| Pieter H.W. | Lubbert | MD PhD | Ziekenhuis Tjongerschans | Heerenveen |
| Francesco U.S. | Mattace‐Raso | MD PhD | Erasmus MC | Rotterdam |
| Marieke C. | Meinardi | MD | Albert Schweitzer Ziekenhuis | Dordrecht |
| Joris J. | Mellema | MD | Ziekenhuis Tjongerschans | Heerenveen |
| Roland M.H.G. | Mollen | MD PhD | Ziekenhuis Gelderse Vallei | Ede |
| Majon | Muller | MD PhD | Amsterdam UMC | Amsterdam |
| Joost C. | Peerbooms | MD | Albert Schweitzer Ziekenhuis | Dordrecht |
| Suzanne | Polinder | PhD | Erasmus MC | Rotterdam |
| Kornelis J. | Ponsen | MD PhD | Noordwest Ziekenhuisgroep | Alkmaar |
| Rudolf W. | Poolman | MD PhD | OLVG | Amsterdam |
| Miruna | Popescu | MD PhD | Noordwest Ziekenhuisgroep | Alkmaar |
| Albert F. | Pull ter Gunne | MD PhD | Rijnstate | Arnhem |
| Bas J. | Punt | MD | Albert Schweitzer Ziekenhuis | Dordrecht |
| Gert R. | Roukema | MD | Maasstad Ziekenhuis | Rotterdam |
| Hilde I.F. | Roijen | MD | Isala | Zwolle |
| Jeanine | Schukking | MD | Rode Kruis Ziekenuis | Beverwijk |
| Josje | Snoek | MD | Bernhoven | Uden |
| Jeroen | Steens | MD PhD | Dijklander Ziekenhuis | Hoorn |
| Charles T. | Stevens | MD | Bernhoven | Uden |
| Dieneke | Van Asselt | MD PhD | Radboud UMC | Nijmegen |
| Romke | Van Balen | PhD | Amsterdam UMC | Amsterdam |
| Esther | Van de Glind | MD PhD | Alrijne Ziekenhuis | Leiderdorp |
| Cornelis L.P. | Van de Ree | MD | Elisabeth‐TweeSteden Ziekehuis | Tilburg |
| Alexander H. | Van der Veen | MD PhD | Catharina Ziekenhuis | Eindhoven |
| Detlef | Van der Velde | MD PhD | St. Antonius Ziekenhuis | Nieuwegein |
| Bart A. | Van Dijkman | MD | Flevoziekenhuis | Almere |
| Sven H. | Van Helden | MD PhD | Isala | Zwolle |
| Paul J. | Van Koperen | MD PhD | Meander MC | Amersfoort |
| Job L.C. | Van Susante | MD | Rijnstate | Arnhem |
| Romuald | Van Velde | MD | Tergooi | Hilversum |
| M. Remmelt | Veen | MD PhD | St. Antonius Ziekenhuis | Nieuwegein |
| Ralf W. | Vingerhoets | MD | Elisabeth‐TweeSteden Ziekehuis | Tilburg |
| Dagmar I. | Vos | MD PhD | Amphia Ziekenhuis | Breda |
| Judith | Wilmer | MD | Catharina Ziekenhuis | Eindhoven |
| Jasper | Winkelhagen | MD | Dijklander Ziekenhuis | Hoorn |
| Johan F.H. | Wold | MD | Meander MC | Amersfoort |
| Robbert A. | Zandbergen | MD | Rode Kruis Ziekenuis | Beverwijk |
| G. (Bert) | Ziere | MD PhD | Maasstad Ziekenhuis | Rotterdam |
| Rutger G. | Zuurmond | MD PhD | Isala | Zwolle |

All participating hospitals are located in The Netherlands
